# Stable Cavitation-Mediated Delivery of miR-126 to Endothelial Cells

**DOI:** 10.3390/pharmaceutics14122656

**Published:** 2022-11-30

**Authors:** Stephanie He, Davindra Singh, Hossein Yusefi, Brandon Helfield

**Affiliations:** 1 Department of Biology, Concordia University, Montreal, QC H4B 1R6, Canada; 2 Department of Physics, Concordia University, Montreal, QC H4B 1R6, Canada

**Keywords:** gene therapy, miRNA delivery, microRNA-126, microbubbles, sonoporation, passive cavitation detection, ultrasound, endothelial cells, HUVEC, therapeutic angiogenesis

## Abstract

In endothelial cells, microRNA-126 (miR-126) promotes angiogenesis, and modulating the intracellular levels of this gene could suggest a method to treat cardiovascular diseases such as ischemia. Novel ultrasound-stimulated microbubbles offer a means to deliver therapeutic payloads to target cells and sites of disease. The purpose of this study was to investigate the feasibility of gene delivery by stimulating miR-126-decorated microbubbles using gentle acoustic conditions (stable cavitation). A cationic DSTAP microbubble was formulated and characterized to carry 6 µg of a miR-126 payload per 10^9^ microbubbles. Human umbilical vein endothelial cells (HUVECs) were treated at 20–40% duty cycle with miR-126-conjugated microbubbles in a custom ultrasound setup coupled with a passive cavitation detection system. Transfection efficiency was assessed by RT-qPCR, Western blotting, and endothelial tube formation assay, while HUVEC viability was monitored by MTT assay. With increasing duty cycle, the trend observed was an increase in intracellular miR-126 levels, up to a 2.3-fold increase, as well as a decrease in SPRED1 (by 33%) and PIK3R2 (by 46%) expression, two salient miR-126 targets. Under these ultrasound parameters, HUVECs maintained >95% viability after 96 h. The present work describes the delivery of a proangiogenic miR-126 using an ultrasound-responsive cationic microbubble with potential to stimulate therapeutic angiogenesis while minimizing endothelial damage.

## 1. Introduction

Gene therapy is a treatment regime in which a specific cell-function-altering piece of genetic material is introduced into target diseased cells. Typically, DNA, mRNA, siRNA, and anti-sense oligonucleotides are the genetic materials used for this approach, either to restore a specific gene function or to turn off a gene involved in pathogenesis. The first authorized clinical trial for gene therapy was for Gaucher’s disease in 1988 (NCT00001234), a lysosomal dysfunction. Mostly due to the fact that it commonly employs viruses to deliver the genetic material, gene therapy was met with mixed success early on in its history [1] owing to complications with the immune response, off-target side-effects, and unwanted or neutral clinical outcomes [2]. Indeed, the critical challenge toward the advancement of this approach is the delivery method itself, and key advances in this field have brought gene therapy back into the spotlight. Nonviral vector approaches, such as systemically injected liposomal constructs, are generally less immunogenic than their viral counterparts, and they can gain target cell specificity through chemical/biological design. Generally, these vectors are considered less efficient than viral vectors, likely due to the fact that they face the endoluminal border and must escape early endosomes to deliver their payload.

More recently, microRNAs (miRs) have been employed in molecular therapeutics. MiRs are noncoding RNA strands that may bind to multiple mRNA targets and modulate their expressions. Nearly three decades ago, the discovery of dysregulated miR in nematodes [3] allowed insight into a better understanding of disease development in a wide array of pathologies [4,5,6]. The pleiotropic nature of miRs makes for a particularly attractive choice for gene therapy, e.g., in applications of diseases with a multifactorial origin [7]. Indeed, miR therapy is being explored preclinically in many areas, including cancer (e.g., pancreatic [8], breast [9], lung [10], and leukemia [11]) and cardiovascular disease (e.g., cardiac hypertrophy [12], arrhythmia [13], cardiac fibrosis [14], and ischemia [15]).

With regard to the application of therapeutic angiogenesis for ischemia, miR-126 is a potentially potent target. miR-126 is highly expressed in endothelial cells, and feasibility studies have shown its role in modulating angiogenesis by repressing angiogenic repressors, such as PIK3R2 from the Akt pathway and SPRED1 from the ERK pathway, to name a few [16,17]. Indeed, in selecting a potential candidate technique for miR-126 delivery or any other miR that has shown to be a major molecular regulator in cardiovascular disease, there is particular interest in exploring ultrasound-based techniques, as echocardiography is commonly clinically used to diagnose many cardiovascular diseases [18].

Ultrasound-sensitive agents, including clinically employed microbubble contrast agents, provide an exciting alternative to more traditional nonviral vectors. Typically between 1 and 8 μm in diameter, microbubbles remain intravascular and are composed of a thin, flexible lipid monolayer shell and vibrate when exposed to ultrasound [19]. Recent studies have demonstrated that, under specific acoustic conditions, ultrasound-stimulated microbubbles can temporarily alter vascular and cellular permeability, thereby providing an endocytosis-independent pathway for exogenous drug delivery [20,21,22,23]. Indeed, as these microbubbles only vibrate as they pass through an ultrasound beam, their activity is spatially and temporally targeted, thus having the potential to limit off-target therapeutic deposition. 

In microbubble-mediated gene therapy [24], the design of the microbubble is critical to ensure sufficient loading capacity. One such approach is to synthesize microbubbles with a surface charge to offer a means to attach genetic payload on the microbubble surface, shown to deliver genetic material more efficiently than co-injected with neutral microbubbles [25,26]. Such efficiency is conferred by protection against circulating RNases in the bloodstream to increase the half-life of the short RNA strand, as well as increasing the available genetic material around the tissue when it is coupled to a vehicle, as opposed to free floating in the systematic circulation [27]. Additionally, the noninvasive nature of the methodology allows for repeated treatment to further increase gene delivery efficiency, as well as the ability to spatially target tissues of interest with focused ultrasound limits off target responses [28].

Ultrasound-mediated microbubble behavior is commonly separated into two distinct physical regimes [29]. If bubbles are exposed to acoustic pressures above a specific threshold, they can rapidly expand and violently collapse during the compression of the transmit pulse. This behavior, whereby microbubble disruption occurs, is dominated by the inertia of the surrounding fluid and is typically termed inertial cavitation, accompanied by broadband spectral emissions. Indeed, miR-based delivery using ultrasound and microbubbles has been shown via this acoustic regime, which does result in physical membrane perforation and enhanced cellular and vascular uptake of material [30,31,32]. In fact, numerous studies have demonstrated the advantage of delivering miR or miR inhibitors through inertial cavitation (typically referred to as ultrasound-targeted microbubble destruction; UTMD) for specific applications, such as cancer cell ablation [30,33,34]. Other applications of gene delivery through UTMD have shown success in preventing organ rejection [35], treating cardiac hypertrophy [31], and even in promoting angiogenesis [32].

While shown to be an effective strategy, microbubble disruption (e.g., inertial cavitation) may trigger severe, undesired bioeffects, including loss of cell viability [36], hemorrhage, or increased inflammation. 

Another approach to microbubble-mediated ultrasound gene delivery, which has been less explored, is to ensure a more controlled microbubble oscillation regimen. Generally, bubbles driven by low-pressure ultrasound elicit repeated periodic, volumetric vibrations about their equilibrium size, whereby they may oscillate spherically or non-spherically. Under specific acoustic conditions, these vibrations can enhance cell permeability due to local fluid microstreaming and prolonged shear stress [37]. This regime is called stable cavitation and is characterized by distinct harmonic scattered pressure emissions [29]. In addition to limiting potential unwanted bioeffects, the prolonged fluid streaming and physical presence of the microbubble may aid in intracellular therapeutic deposition.

Indeed, as the majority of investigations of ultrasound-mediated gene delivery rely on bubble destruction, the novelty of this work presented here is via our intended goal of demonstrating that modest levels of gene therapy can be achieved using gentle bubble vibrations (stable cavitation without bubble disruption), with potential significance toward the design of repeat treatment paradigms. This entails the synthesis of high-capacity gene-loaded microbubble constructs that are clinically feasible in terms of stability, concentration, nonlinear echo, and gene-loading concentration, followed by confirmation of small but significant gene delivery so as to preserve cell viability and ensure successful modulation of salient downstream protein expression and physiological endpoints. 

In this present study, we aim to investigate ultrasound-mediated gene delivery using stable cavitation, specifically in the context of therapeutic angiogenesis. First, we synthesized and characterized a cationic lipid microbubble formulation decorated with an miR-126 mimic. Next, we demonstrate that these microbubbles can be used to deliver their miR-126 cargo within endothelial cells in a viable manner while undergoing stable cavitation, monitored with passive cavitation techniques. Lastly, we assess the functional and physiological endpoints of the miR-126 delivery.

## 2. Materials and Methods

### 2.1. Contrast Agent Microbubble Preparation

Cationic phospholipid-encapsulated microbubbles were synthesized via a modification of an existing in-house formulation [38]. Briefly, the microbubbles were prepared from a lipid aqueous dispersion composed of 1,2-distearoyl-sn-glycero-3-phosphocholine (DSPC; Avanti Polar Lipids, Alabaster, AL, USA), polyoxyethylene (40) stearate (PEG40S; Sigma-Aldrich, St. Louis, MO, USA), and 1,2-stearoyl-3-trimethylammonium-propane (DSTAP; Avanti Polar Lipids, Alabaster, AL, USA). DSPC, PEG40S, and DSTAP (0.41:0.50:0.09) were dissolved in a solution buffer consisting of PBS-EDTA (1 mM), propylene glycol and glycerol (0.80:0.15:0.05) at a concentration of 4 mg/mL. The lipid solution was then dissolved in a 20 kHz ultrasonic bath (Branson Ultrasonics, Danbury, CT, USA) at 65 °C until a homogenous clear solution was observed. Samples of microbubbles were formed by aliquoting 1.5 mL of this solution in glass vials and sealed with a rubber stopper. The air from the vial was removed via vacuum and replaced with perfluorobutane (C_4_F_10_; FluoroMed, Round Rock, TX, USA). To synthesize the microbubbles, vials were allowed to reach room temperature to ensure consistent size distribution and scattering activity [39], subjected to mechanical agitation using the VialMix™ (Lantheus, Billerica, MA, USA) for the standard 45 s period, and then were set to cool down to room temperature for 15 min. Vials were then decanted for 8 min to ensure the removal of large microbubbles, and bubbles were withdrawn using a 1 inch 19G needle, along with another 19G needle for venting purposes. Agent was consistently extracted from just below the rubber stopper to reduce population variability. After decantation, 0.7 mL was extracted from the glass vial and the microRNA-126 was added to the microtube for incubation. Microbubbles were washed as per described in Wang et al. [25]. Briefly, the mixture was diluted to 1 mL with DEPC-treated diH_2_O and centrifuged at 400× *g* for 3 min in a 3 mL syringe. The bottom 0.5 mL was discarded to remove the smaller microbubbles, and the remainder of the solution was diluted in DEPC-treated diH_2_O to fix the concentration to 10⁹ microbubbles/mL for the experiments. 

### 2.2. Microbubble Characterization

The size distribution, concentration, and stability of these in-house microbubbles were quantified using a Coulter Counter (Multisizer 4e; Beckman Coulter, CA, USA). Microbubbles were diluted 1:1000 in ISOTON II (Beckman Coulter) and sampled using the 30 μm aperture, which enables an effective measurable size range of 0.6–18 μm. For a given vial, measurements were repeated every 20 min for up to 80 min post activation to assess microbubble stability. For a subset of experiments, the zeta potential was measured using a Zetasizer Nano ZS (Malvern Panalytical, Malvern, UK). Microbubbles were diluted to a 0.2% solution within diH_2_O, and measurements were taken within 10 min of having extracted the agent from the vial. For each of these measurements, at least *n* = 3 vials were used for statistical purposes. 

To confirm that our agent is acoustically active, the echogenicity of the microbubbles was measured with a clinical ultrasound system (model iU22 Philips Healthcare, Andover, MA, USA) using a C5-2 probe. The microbubbles were diluted 10,000-fold in gas-equilibrated diH_2_O and fed into a wall-less 0.8 cm diameter cardiac Doppler flow phantom (model 523A, ATS Laboratories, Norfolk, VA, USA) at a velocity of ~14–16 mm/s. The depth was fixed at 8 mm, and three videos were recorded in B mode and contrast mode for 2 min. The signal-to-noise ratio (SNR) was plotted ± the standard deviation at different timepoints following microbubble extraction from its vial.

### 2.3. miR-126 Loading Protocol and Characterization

To confirm gene coupling to the outer shell of our cationic microbubbles, a solution of microbubbles was incubated at room temperature for 15 min with a red fluorescent siRNA (Alexa Fluor 555 BLOCK-iT, Thermo Fisher Scientific, Waltham, Brea, MA, USA) in a 1:1 proportion. These microbubbles were visualized using an RFP light cube from the EVOS M7000 imaging system (Thermo Fisher Scientific). After confirming the cationic nature of our microbubbles, we aimed to load our gene of interest miR-126 (hsa-miR-126-3p; sequence: UCGUACCGUGAGUAAUAAUGCG; Thermo Fisher Scientific). 

To quantify miR-126 loading capacity, the microbubbles were incubated with increasing miR-126 concentrations (miR-126 input) at room temperature for 15 min on a tube revolver rotator. These concentrations were fixed from 1 µg miR-126/10⁹ microbubbles to 20 µg/10⁹ microbubbles, where 20 µL of a RNA loading dye [40] was added to equal volume of the miR-126 and microbubbles solution, and loaded in a 12.5% (19:1 acrylamide/bis-acrylamide) (Thermo Fisher Scientific) urea PAGE [40]. A control lane where equal amounts of miR-126 was diluted in DEPC-treated diH_2_O was also loaded next to each miR-126 concentration tested. We ran the gel at 65 V until separation of bromophenol blue (Sigma) and xylene cyanol (Thermo Fisher Scientific). The gel was stained with a 1:10 000 solution of SybrGreen II RNA gel stain (Thermo Fisher Scientific) in TBE buffer for 15 min. The gel was imaged with on a G:BOX F3 (Syngene, Cambridge, UK), and the bands were analyzed on ImageJ (U.S. National Institutes of Health, Bethesda, MD, USA). We assumed that the bands observed corresponded to the unbound miR-126 (when loaded with microbubbles) and were compared to the control bands (miR-126 with diH_2_O), which resulted in the percentage of free miR-126 in the gel.

(1) $$\%free\;=\;\frac{band\;intensity\;value\;of\;miR\;-\;126\;with\;microbubbles}{band\;intensity\;value\;of\;miR\;-\;126\;in\;diH_{2}O}.$$

Lastly, the percentage of miR-126 bound was obtained with the following equation:

(2) % bound = %100 − % free

where we report here the mass of loaded miR-126 as

(3) mass of bound miR − 126 = % bound × mass of miR − 126 input.

These data points were plotted to find the plateau to determine the maximum achievable mass of bound miR-126 per 10⁹ microbubbles.

### 2.4. Cell Culture

Primary human umbilical vein endothelial cells (HUVECs; No. C2519A, Lonza, Verviers, Belgium) were cultured in medium (EGM-2, No. C3162, Lonza, Belgium) and incubated at 37 °C and 5% CO_2_. For experiments, the cells were harvested with a 0.05% solution of trypsin-EDTA (Wisent, QC, Canada) at 90% confluency and placed in a suspension within cell culture medium at a concentration of 500 k cells per mL. All experiments were performed on HUVECs characterized by a passage number between 3 and 10.

### 2.5. Ultrasound Apparatus and Experimental Procedure

**Figure 1:**
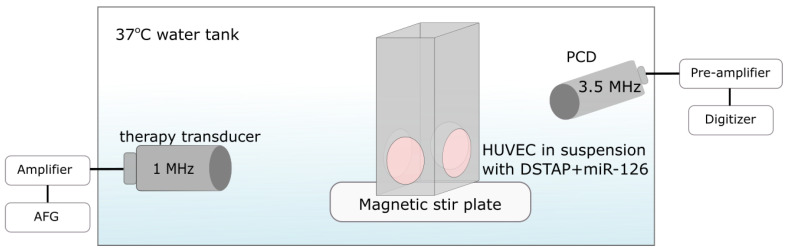
**Experimental setup equipped with two co-aligned transducers for ultrasound-mediated gene delivery to endothelial cells**. The therapy transducer (1 MHz) focuses within a cell suspension chamber, while the second transducer (3.5 MHz) is used for passive detection of bubble echoes.

We employed a custom-designed ultrasound treatment tank in order to assess microbubble-mediated miR-126 delivery to endothelial cells (Figure 1). The treatment tank was a 30 L plastic container. It was filled with 15 L of gas-equilibrated diH_2_O, enough volume to submerge both the transducers, but kept below the opening of the sample chamber. The water temperature was maintained at 37 °C using an immersion heater circulator (VWR model 1120, Radnor, PA, USA). The tank consisted of a sample chamber and two co-aligned single-element transducers. A magnetic stir bar was placed inside the sample chamber, which is made of acrylic and sealed with mylar windows (25 µm thickness) to allow for ultrasound transmission. The unit was placed on a magnetic stir plate. Samples were prepared by incubating miR-126-loaded microbubbles with HUVECs at a fixed ratio of 50 bubbles per cell. Placed within our warmed sample chamber, this cocktail was constantly mixed using a magnetic stir bar to ensure homogeneous distribution of the microbubbles and cells. After a 1 min wait to ensure equilibrium, the sample was treated with ultrasound. The treatment transducer (1 MHz, f = 25.4 mm, f# = 1.33) was driven at 1 MHz with 1000 cycles, varying pulse repetition intervals from 2.5–5 ms (duty cycles of 20–40%) at a peak negative pressure of 52 kPa generated from an arbitrary function generator (AFG31000, Tektronix, Beaverton, OR, USA) and amplified using an RF power amp (model 105A100B, Amplifier Research, Souderton, AR, USA) for a treatment duration of 2 min. The acoustic pressure was measured in free space within a separate water tank using a ‘bullet’ hydrophone (HGL-0200, ONDA, Sunnyvale, CA, USA). The second transducer (3.5 MHz flat transducer, Olympus) was used as a passive cavitation detector to record microbubble scattering, specifically, to assess the presence of stable and inertial cavitation. Receive echoes were amplified (AU-1579, 0.7–200 MHz, MITEQ, Hauppauge, NY, USA), bandpass-filtered, and then digitized (Gage Razor Express CompuScope, Lockport, IL, USA) for off-line analysis using custom MATLAB software (Mathworks, Natick, MA, USA). The frequency-dependent transfer function of this receive circuit was not determined; thus, all measurements are relative. Joint time–frequency analysis was performed with a window size of 50 ms and a 90% overlap. A Hamming window was applied to the RF data prior to obtaining the fast Fourier transform (FFT). 

Following ultrasound treatment, the cells were transferred from the custom-designed suspension chamber to a conical tube left at room temperature for 5 min to allow for sonoporation recovery. These cells were then placed on ice until all the samples were treated before washing three times at 220× *g* for 5 min at 4 °C per wash to remove the remaining bubbles. The sham controls were handled similarly without ultrasound. 

### 2.6. RT-qPCR 

Immediately following the washes, the total RNA content of the cells was extracted using a mirVana™ miRNA isolation kit (Invitrogen), and 10 ng RNA samples were used for PCR. The primers were all purchased from Thermo Fisher Scientific, and, although their sequences are proprietary information, their product numbers (PN) are listed. The PCR primers used to generate cDNA were U6 (PN: 4440887 RT 001973) and has-miR-126 (PN 4427975 39G01 RT 002228) on a thermal cycler (Applied Biosystems, Waltham, MA, USA) set at 16 °C for 30 min, 42 °C for 30 min, and 85 °C for 5 min. RT-qPCR was performed using TaqMan™ Fast Advanced Master Mix no UNG (Thermo Fisher Scientific) on a QuantStudio™ 3 (Thermo Fisher) set at 95 °C for 20 s, then 40 cycles of 95 °C (1 s) to 60 °C (20 s). The primers used for RT-qPCR were U6 (PN: 4440887 TM 001973) and miR-126 (PN: 4440887 74A03 TM 002228). Relative miR-126 levels were calculated using the 2(−∆∆Ct) method using U6 as the housekeeping control. 

### 2.7. Viability Assay

Endothelial cell viability was measured after 2 days and 4 days, whereby 20,000 and 10,000 cells were seeded on 96-well dishes in triplicate. After washing the cells with PBS three times, MTT reagent (Sigma) was added as per the manufacturer’s protocol. The absorbance was measured on a Varioskan™ LUX microplate reader (Thermo Fisher Scientific) at 540 nm after a 4 h incubation in 37 °C in 5% CO_2_ and solubilizing the formazan with DMSO (BioRad, Hercules, CA, USA) for 10 min. Viability was assessed relative to sham controls. 

### 2.8. Western Blotting

Following the washes after ultrasound treatments, cells were seeded on six-well dishes and harvested 2 days later. Total protein was extracted using RIPA lysis buffer (Alfa Aesar, Haverhill, MA, USA), and 20–30 µg of protein was loaded per lane in a 7.5% acrylamide (37.5:1 acrylamide/bis-acrylamide) (BioRad) SDS-PAGE, 1.5 mm thick. We let the gel run at 70 V for 30 min and 100 V until complete separation of the PageRuler™ Plus prestained protein ladder (Thermo Fisher Scientific). The proteins were transferred on a PVDF membrane overnight in Towbin buffer at 4 °C. The membrane was stained with 0.5% Ponceau S (Sigma) in 1% glacial acetic acid for 5 min to ensure proper protein transfer and destained with TBS. Following blocking in TBS with 5% BSA (Wisent) for 1 h at room temperature, the PVDF membrane was cut in three sections horizontally to separate the target proteins according to their molecular weights. 

The antibodies used for blotting were monoclonal anti-SPRED1 (Santa Cruz Biotechnology, Dallas, TX, USA), monoclonal anti-PI-3-kinase p85β (Santa Cruz Biotechnology), and anti-GAPDH (Invitrogen), diluted in TBST-2% BSA for 1 h at room temperature. The first section was cut at the 70 kDa band to probe the PIK3R2. The second section was cut around the 40 kDa band (between the 35 kDa and 55 kDa bands) to probe the SPRED1 antibody. The last section was used to probe GAPDH. When we could not obtain a satisfactory separation from ladder to cut the membrane between the 55 kDa and 35 kDa bands, the membrane was only cut at the 70 kDa band. GAPDH was then probed first, stripped with a mild stripping buffer (Abcam) for 5 min, and washed with PBS and TBS for 5–10 min twice each. Blocking could then be repeated on this half membrane to probe SPRED1. The membrane was then incubated in with goat anti-mouse IgG (H + L) HRP (Invitrogen) secondary antibody diluted in TBST-2% BSA for 1 h at room temperature. PVDF blotted membranes were labeled with Pierce ECL Western Blotting Substrate (Thermo Fisher Scientific) prior to imaging on an Amersham Imager 600 (GE Healthcare, Chicago, IL, USA). Bands were analyzed in ImageJ. 

### 2.9. Endothelial Tube Formation Assay

A growth factor-reduced Matrigel^®^ matrix basement membrane (Corning, NY, USA) was prepared on a 96-well dish according to the manufacturer’s protocol. Following the washes, 2000 cells were seeded atop the Matrigel for 16 h. HUVECs were then stained with Calcein-AM (Thermo Fisher) at 1 µg/mL and visualized on an epi-fluorescence microscope at 488 nm (EVOS system, Life Technologies). Angiogenesis quantification was assessed by AutoTube [41,42,43] on MATLAB.

## 3. Results

### 3.1. Characterization of DSTAP Microbubbles and miR-126 Loading

**Figure 2:**
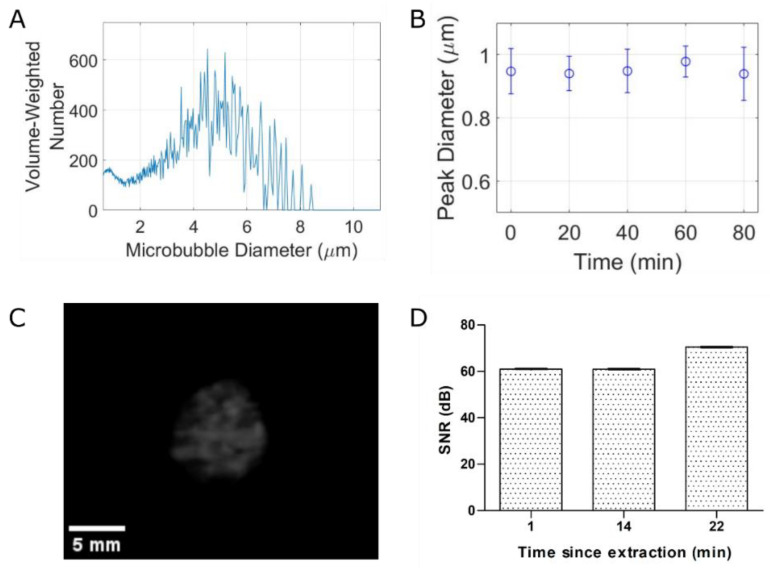
**Our stable microbubble formulation can be used for clinical contrast imaging.** (**A**) Volume-weighted size distribution with a concentration of (1.01 ± 0.31) × 10⁹ bubbles/mL. (**B**) Stability over 80 min. (**C**) Nonlinear contrast imaging of microbubbles in wall-less tissue phantom. (**D**) SNR analysis of contrast signal over 22 min post-agent extraction.

The microbubble size distribution is shown in Figure 2A, depicting a polydisperse population with a peak volume-weighted diameter of 4.56 ± 0.32 µm. Stability tests, where measurements were taken every 20 min, suggest nonsignificant (*p*-value = 0.313, *n* = 3) changes in microbubble diameter (Figure 2B) and concentration (1.01 × 10⁹ ± 0.31 × 10⁹ microbubbles/mL) over 80 min. The size and concentration information reported here is consistent with clinically employed agents [44,45,46]. 

Clinical contrast echo from the agent—which is indicative of nonlinear scattering of the microbubbles—is shown qualitatively in Figure 2C and quantified in Figure 2D. The large contrast signal-to-noise ratio of the agent remains relatively constant up to 22 min following agent extraction from the sealed vial (62.95 ± 2.56 to 70.24 ± 2.76 dB). These data, along with the concentration and stability properties, suggest its viability as a clinical agent.

**Figure 3:**
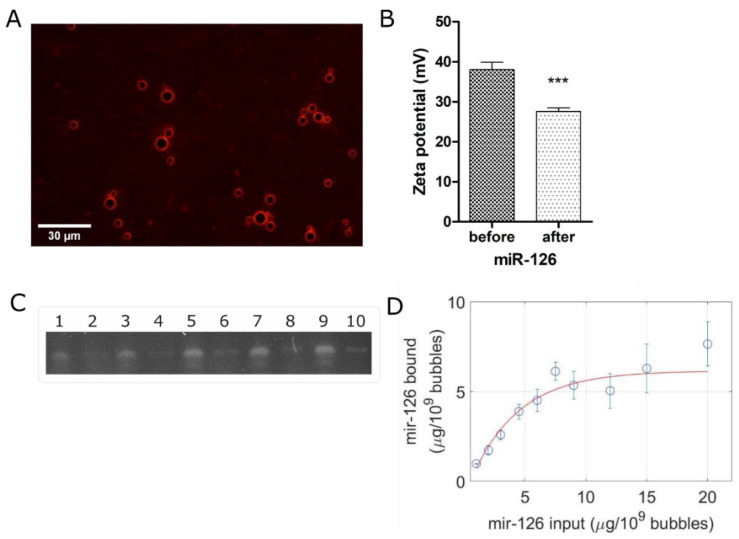
**Gene coupling characterization results in 6 µg of miR-126 per 10^9^ microbubbles.** (**A**) Fluorescent siRNA localization on microbubble surface. Scale bar: 30 µm. (**B**) Surface charge before and after coupling of 6 µg of miR-126 per 10^9^ microbubbles. (**C**) Acrylamide/urea PAGE of miR-126 binding capacity on DSTAP: odd-numbered lanes show free miR-126 in DEPC H_2_O and even-numbered lanes show unbound miR-126 after incubation with DSTAP. (**D**) Quantification of miR-126 binding capacity on DSTAP. (***: *p* < 0.001).

In order to test the cationic nature of this in-house agent, as well as to provide evidence for gene coupling along the surface of the microbubbles, Figure 3A highlights the surface distribution of a surrogate fluorescence siRNA. It can be seen from this micrograph that the fluorescent nucleic acid conforms to the outer surface (i.e., the shell) of the microbubbles. Furthermore, the cationic nature of our agent was confirmed via assessment of the surface charge, which was determined to be +38.04 mV pre miR loading, decreasing to +27.60 mV post miR loading (Figure 3B; *p* < 0.0002). To quantify this miR-126 carrying capacity, gel electrophoresis was performed to compare the amount of free-miR-126 in diH_2_O compared to unbound miR-126 from microbubbles at varying quantities of miR-126 (Figure 3C). Binding capacity was determined from plotting a saturation curve (red) and the value at which the curve plateaus is defined as the binding capacity. For our formulation, we determined a loading capacity of 6 µg of miR-126 per 10^9^ microbubbles (Figure 3D), which is what we used for all subsequent experiments.

### 3.2. Ultrasound-Mediated miR Delivery Using miR-126-Conjugated DSTAP Microbubbles

**Figure 4:**
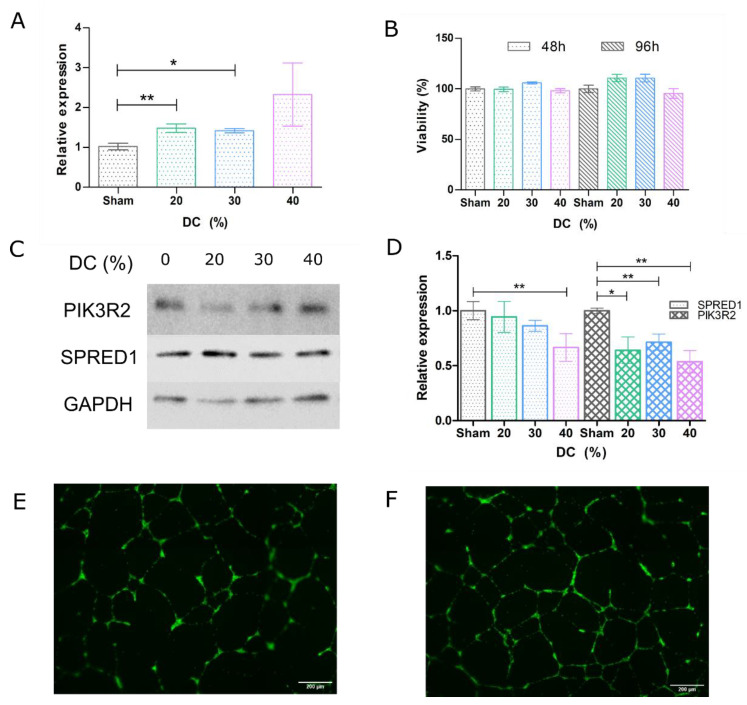
**Ultrasound-mediated miR-126 delivery results in viable physiologically relevant treatment of endothelial cells.** (**A**) RT-qPCR analysis of the relative miR-126 expression from treated HUVECs. (**B**) Viability assessment of the cells 48 h and 96 h following ultrasound treatment by MTT assay. (**C**) PIK3R2 and SPRED1 protein expression in treated HUVECs determined by Western blotting. (**D**) Aggregate relative PIK3R2 and SPRED1 expression 48 h following HUVEC treatment. (**E**) HUVEC tube formation assay, untreated and (**F**) treated at 20% DC. In aggregate, the tube network increased by 22–26% and exhibited a 5–17% increase in branching nodes. Scale bar: 200 µm. (*: *p* < 0.05, **: *p* < 0.01). Grey: sham, green: 20% DC, blue: 30% DC, magenta: 40% DC.

Ultrasound-assisted viable miR-126 delivery to endothelial cells via stable cavitation was demonstrated in Figure 4. Figure 4A indicates a gradual increase in miR-126 levels, from 1.482 to 2.326 relative to the sham control, with increasing duty cycle (*n* = 3 to 8, *p*-values = 0.004, 0.02, 0.08, respectively). Our data indicate that, under this ultrasound treatment regimen, endothelial cell viability is maintained (>95%) (Figure 4B; *n* = 6 to 24, *p*-value nonsignificant). Further, as confirmation of functional delivery, we assayed the protein levels of miR-126 target proteins SPRED1 and PIK3R2. These were downregulated 2 days following gene delivery; we observed a 4% to 33% decrease in SPRED1 with increasing duty cycle (*n* = 3 to 4; *p*-value = 0.0345 at duty-cycle of 40%), while PIK3R2 decreased from 36% to 46% with increasing duty cycle (Figure 4D; *n* = 3–4; *p*-values = 0.02, 0.008, and 0.003, respectively). 

A second subset of treated cells were used to grow on Matrigel basement membrane matrix, where endothelial tube formation was assessed 16 h after seeding. As can be seen in the representative images shown in Figure 4E,F, tubule networks were significantly more complex in miR-126-treated endothelial cells compared to sham control (Figure 4E). Specifically, tube network increased by 22–26% and exhibited a 5–17% increase in branching nodes.

### 3.3. Passive Cavitation Detection

**Figure 5:**
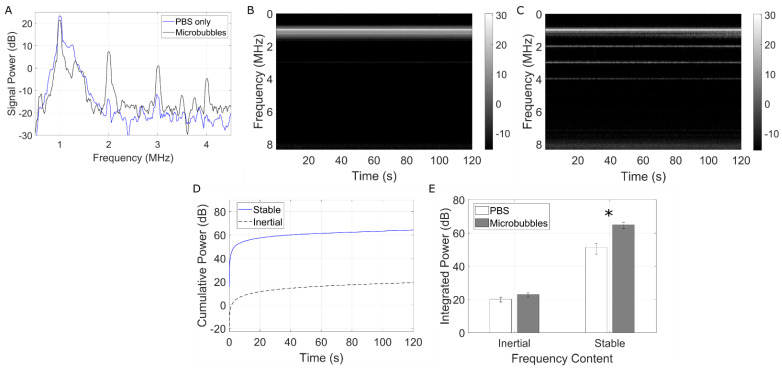
**Microbubbles undergo stable cavitation during gene delivery treatment.** (**A**) The microbubble echo spectrum at the beginning of treatment (black line) as compared to a PBS only control (blue line). (**B**) Time-frequency plots of the resulting cavitation echoes in PBS (control) and (**C**) miR-126 loaded microbubbles. This time-frequency depiction clearly shows prolonged harmonic content (stable cavitation) throughout the 2 min treatment, and the absence of any broadband spectral emissions (inertial cavitation). (**D**) Quantification of the cumulative spectral power from the stable (blue, solid line) and inertial (black, dashed-line) cavitation frequency bands over the total treatment time of a given sample. (**E**) Quantification over all samples compared to PBS only controls. Asterisks denote statistical significance (*p* < 0.02).

To confirm stable microbubble activity during treatment, the gene delivery setup (Figure 1) was concurrently equipped with a passive cavitation detection transducer. Figure 5A shows a representative example of the frequency spectrum at the beginning of the 2 min treatment with microbubbles (30% duty cycle; black) overlaid with pure PBS (blue). It can be clearly seen that, under this acoustic regime, microbubbles underwent stable cavitation, as confirmed by narrowband signal power peaking at second, third, and fourth harmonics (2, 3, and 4 MHz) and an absence of broadband emissions. Furthermore, time–frequency analysis (Figure 5B,C) confirmed that microbubbles were undergoing stable cavitation throughout the entire duration of the ultrasound treatment. Figure 5D depicts the cumulative spectral power from the stable (blue, solid line) versus inertial (black, dashed line) cavitation frequency bands for a given sample. Figure 5E quantifies the total integrated power for microbubble samples and PBS control, highlighting the virtual absence of inertial cavitation and a statistically significant 13.5 dB in stable cavitation activity (*p* < 0.02). 

## 4. Discussion 

In this study, we developed a methodology to deliver modest amounts of miR-126 to endothelial cells for the purpose of therapeutic angiogenesis. Our results demonstrate that miR-126 can be successfully delivered intercellularly via ultrasound and microbubbles in sufficient quantities to elicit downstream regulation of target proteins and a viable physiological response.

It is perhaps worth noting that one of the limitations of the present study is that the endothelial cells were treated in suspension, as opposed to within the more biologically relevant monolayer configuration. This could have negatively affected transfection efficiency because the cell morphology of these cells in suspension exhibited less surface area as opposed to their adherent morphology and, incidentally, were less likely to interact with a miR-126-decorated microbubble in the cell chamber. To address this in future work, another nonadherent cell line could be investigated, such a endothelial progenitor cells [47], or cells could be cultivated and treated under fluidic conditions more accurate mimic a vessel [48]. This arrangement, however, was chosen in order to position two co-aligned transducers in such a way as to be able to passively record the acoustic emissions during treatment (Figure 5). This is essential as it provides evidence of microbubble stable cavitation. Due to the specific ultrasound parameters employed (e.g., long pulses), this would have been challenging to conduct using a single transducer with active listening (pulse-echo). 

Indeed, while designing the ultrasound regimen, we focused on maintaining cellular viability albeit at the expense of lower transfection efficiency in order to substantiate the feasibility of microbubble-mediated gene delivery as opposed to other gene delivery methods. For instance, in peptide-mediated miR-126 delivery to vascular endothelial cells, Zhou and colleagues reached a 3.5-fold increase in miR-126 but reported cell death up to 20% at the highest peptide dose [49]. Indeed, by devising a microbubble-mediated miR-126 delivery treatment scheme that minimizes cell death, this enables the potential for repeat treatments to further increase the transfection efficiency and enhance the effects of the genetic payload [18,50]. 

Microbubble-mediated gene therapy, whether employing miRs or other nucleic acid payloads, has been demonstrated in cancer and cardiovascular applications through cavitation regimes in which the bubbles undergo disruption (e.g., ultrasound-targeted microbubble disruption; UTMD [27]). Violent microbubble collapse initiated this way requires strong acoustic forcing conditions, typically large peak-negative pressures [29]. While this technique has shown promising results in gene deposition within the target tissue [51,52,53], the bubble involution process can initiate high-speed liquid jets along with other cavitation-based phenomena that may lead to localized endothelial bio-effects. Endothelial cell damage and denudation have been reported in both microvessels [54,55] and larger vasculature, events which have been correlated to excessive inertial cavitation doses [54] (a surrogate measure of inertial cavitation and bubble disruption [29]). Further, microvascular hemorrhage and red blood cell extravasation associated with violent microbubble collapse have also been observed [56,57,58,59], and recent reports have demonstrated the potential for inertial cavitation to trigger endothelial apoptosis pathways [60]. As a consequence, our treatment paradigm consists of a very low peak negative pressure and large number of cycles, so as to encourage miR payload release from the cationic microbubble surface by stable cavitation—similar in principle to other drug-loaded microbubbles [61]. Indeed, as gene delivery was achieved in the absence of broadband emissions and via these long duration pulses, our results suggest that sustained gentle microbubble vibrations, radiation forces, and agitation lead to delivery of miR-126, a combination that was persistent throughout the 2 min treatment time (see Figure 5). The hypothesized drug delivery mechanism here is two-stage. Firstly, long pulse-driven microbubbles can initiate prolonged lipid and cargo release as compared to those driven by shorter pulses at a given pressure amplitude [62]. Secondly, microbubble translation and constant agitation decrease the average microbubble–cell distance, which both aids in microbubble-mediated cell membrane perforation [63] and lowers the distance over which the free miR mimic is required to diffuse to enter the neighboring cell. It is also of interest to note that ultrasound has been shown to modulate endocytosis activity [64,65]; however given the timescales of the miR-delivery performed here, it is perhaps not likely to be the dominant mechanism [23]. 

## 5. Conclusions

We synthesized a miR-126-bearing microbubble agent with a gene loading capacity of 6 μg per 10^9^ bubbles, characterized by a bubble concentration, size distribution, and stability similar to that of currently used clinical agents. Using a low-pressure, long-pulse acoustic regime, we were able to show delivery of up to 2.3× in miR-126 to endothelial cells compared to sham controls while maintaining cell viability, resulting in the expected physiological behavior, including downregulation of angiogenic suppressor proteins SPRED1 and PIK3R2. Furthermore, simultaneous passive cavitation detection confirms that this was a stable cavitation treatment, thus minimizing potential damage caused by more violent, inertial cavitation approaches. 

## Data Availability

Not applicable.
